# Clinical efficacy of antibacterial drugs combined with Proton Pump Inhibitors and Bismuth in the treatment of Helicobacter Pylori-infected Peptic Ulcer

**DOI:** 10.12669/pjms.41.11.12314

**Published:** 2025-11

**Authors:** Xiaoying Fu, Qiyu Shi, Yan Jiao

**Affiliations:** 1 Xiaoying Fu Department of Medicament, Cangzhou Medical College, Cangzhou 061001, Hebei, China; 2 Qiyu Shi Gastrointestinal Endoscopy Diagnosis and Treatment Center, Cangzhou People’s Hospital, Cangzhou, 061001, Hebei, China; 3 Yan Jiao Drug Analysis Teaching and Research Section, Cangzhou Medical College, Cangzhou 061001, Hebei, China

**Keywords:** Helicobacter pylori Infection, Peptic Ulcer, Antibacterial Drugs, Proton Pump Inhibitors, Bismuth Agents, Clinical Effect

## Abstract

**Objective:**

To investigate the clinical efficacy of antibacterial drugs combined with proton pump inhibitors and bismuth in the treatment of Helicobacter pylori (Hp)-infected peptic ulcer.

**Methodology:**

This was a retrospective study. A total of 116 patients with Hp-infected peptic ulcer treated at Cangzhou People’s Hospital from January 2023 to December 2024 were selected as study subjects and randomly divided into an observation group(n=58) treated with antibacterial drugs in addition to the treatment for the control group and a control group(n=58) received proton pump inhibitors and bismuth using the computer-based lottery method. The clinical efficacy, time to symptom subsidence treatment safety were assessed and compared between the two groups.

**Results:**

The overall response rate of the observation group was higher than that of the control group(*P*<0.05). Specifically, the time to symptom subsidence in the observation group was shorter than that in the control group(*P*<0.05), and the post-treatment levels of G-17, PGI and PGII in the observation group were lower than those in the control group(*P*< 0.05). Meanwhile, the post-treatment serum levels of GAS, VIP, and MOT in the observation group were higher than those in the control group(*P*<0.05). Additionally, the observation group reported lower post-treatment serum levels of TRX-1, TRAF1, and MMP-9 than the control group(*P*<0.05), with no significant differences observed in the incidence of adverse reactions between the two groups(*P*>0.05).

**Conclusion:**

Antibacterial drugs combined with proton pump inhibitors and bismuth are remarkably effective in treating Hp-infected peptic ulcer, which promotes the subsidence of symptoms, improves gastric function, increases gastrointestinal hormone levels.

## INTRODUCTION

Helicobacter pylori(Hp) is a Gram-negative bacterium that primarily colonizes the gastric mucosa of patients, which proliferates extensively in the stomach and causes infections, followed by damaging the gastric mucosal tissue, ultimately resulting in conditions such as chronic gastritis, gastric ulcers, and even gastric cancer.^1^,^2^ The incidence of Hp infection is notably high clinically, with incomplete statistics indicating a prevalence of up to 50% for Hp infection among adults in China.^3^,^4^ As a significant harm induced by Hp infection, peptic ulcers refer to the condition where the inner walls of the stomach and duodenum are damaged, leading to ulceration, with patients often experiencing upper abdominal pain, acid reflux, and, in severe cases, bleeding or perforation, threatening their life and health.^5^

The treatment for Hp-infected peptic ulcers primarily involves proton pump inhibitors (PPIs) and bismuth. Specifically, PPIs exhibit a strong acid-suppressing effect, which reduces gastric acid secretion, promotes gastric mucosal healing, and exhibits anti-Hp activity. Bismuth, on the other hand, is a commonly used gastric mucosal protectant that prevents gastric acid and food contents from eroding the ulcerated mucosa. However, with the increasing clinical application of these treatments, the application of PPIs and bismuth alone has demonstrated insufficient efficacy, particularly regarding the effectiveness against Hp infection, leading to the recurrence of disease in patients.^6^

Given this, the treatment efficacy for patients with Hp-infected peptic ulcers needs to be enhanced by adding appropriate medications to the conventional treatment regimen. In this respect, the selection of antibacterial drugs that are effective against Hp to strengthen the antibacterial effect is considered a new approach to treating Hp-infected peptic ulcers.^7^ Therefore, this study is designed to evaluate the clinical efficacy of the antibacterial drug amoxicillin combined with PPIs and bismuth in patients with Hp-infected peptic ulcers.

## METHODOLOGY

This was a retrospective study. A total of 116 patients with Hp-infected peptic ulcers admitted to the Cangzhou People’s Hospital from January 2023 to December 2024 were selected as subjects and were randomly divided into an observation group (n=58) and a control group (n=58) using the computer-based lottery method. The observation group: 37 males and 21 females, aged 18-62 years (average: 42.68±8.21 years), with a BMI of 18-27 kg/m² (average: 22.18±2.81 kg/m²) and a duration of illness of 3-11 months (average: 7.07±1.90 months). The control group: 35 males and 23 females, aged 19-65 years (average: 43.82±7.89 years), with a BMI of 18-27 kg/m² (average: 22.25±2.85 kg/m²) and a duration of illness of 3-11 months (average: 7.12±1.92 months). No significant differences were observed in general data between the two groups (*P*>0.05).

### Ethical Approval:

The study was approved by the Institutional Ethics Committee of Cangzhou Medical College (No.:20240326; Date: March 26, 2024), and written informed consent was obtained from all participants.

### Inclusion criteria:

- Patients diagnosed with peptic ulcer through gastroscopy, meeting the criteria outlined in the “Guidelines for the Diagnosis and Treatment of Peptic Ulcers (2023)”.^8^
- Patients diagnosed with Hp infection through the C13 breath test.
- Patients without contraindications to treatment with antibacterial drugs, proton pump inhibitors, bismuth, etc.
- Patients provided informed consent to the treatment regimen.
- The feasibility of the plan was approved by an ethics review.

### Exclusion criteria:

- Patients with other digestive system diseases.
- Patients with nausea or tumors.
- Patients with liver or kidney failure.
- Patients who received other treatment plans simultaneously.
- Patients who did not complete the treatment.
- Patients with false positive HP infection.

### Treatment Regimen:

The control group was treated with PPIs and bismuth by taking Rabeprazole enteric-coated capsules (Zhuhai Rundu Pharma, specification: 10 mg, GYZZ H20050228) orally, 10 mg each time, twice daily, and bismuth potassium citrate capsules (Dawnrays Pharmaceutical (Holdings) Limited, specification: 0.3 g, GYZZ H10983185) orally, 0.6 g each time, twice daily. The observation group received antibacterial treatment in addition to the same basic therapy as the control group by taking amoxicillin capsules (Beijing QWT Pharmaceutical, specification: 0.25 g, GYZZ H11022520) orally, 1.0 g each time, twice daily. Both groups were treated for 14 days.

### Evaluation Criteria:

- *Comparison of Clinical Efficacy:* The standards refer to the “Expert Consensus on the Prevention and Control of Helicobacter pylori Infection in Chinese Residents (2021)”.^9^ Significantly Effective: All symptoms of the patients were basically resolved, and Hp is negative; Effective: Symptoms are significantly alleviated, and Hp is negative upon re-examination; Ineffective: Symptoms are not improved, and Hp is positive. Overall Response Rate = Significantly Effect % + Effective %.
- *Comparison of Time to Symptom Subsidence:* The time taken for the subsidence of symptoms such as abdominal pain, subsidence of bleeding, reduction of bloating and belching, and healing of ulcer wounds in both groups were recorded.
- *Comparison of Gastric Function Indicators:* 3-ml of fasting venous blood was collected from patients and centrifuged with an ATC-12R centrifuge (Shanghai Dove Medical Equipment Co. Ltd., HXZZ 2018009123). Afterward, the levels of Gastrin-17 (G-17), Pepsinogen I (PG I), and Pepsinogen II (PG II) were detected using an FH-400B automatic biochemical analyzer (Shanghai Fenghui Medical Instrument Co., Ltd., HXZZ 2019123148) . The detection kits were purchased from Qingdao Sankai Science & Tech Co., Ltd.
- *Comparison of Gastrointestinal Hormone Levels:* The aforementioned serum samples were tested for serum levels of Gastrin (GAS), Vasoactive Intestinal Peptide (VIP), and Motilin (MOT) through the enzyme-linked immunosorbent assay (ELISA). The detection instrument was a TPO-90 ELISA reader (Beijing Hangyu Langqin Medical Equipment Co., Ltd., JXZZ 20152400031), and the detection kits were purchased from Zhejiang Kante Biotechnology Co., Ltd.
- *Comparison of Inflammatory Factor Levels:* The aforementioned serum samples were analyzed for serum levels of Thioredoxin-1 (TRX-1), Tumor Necrosis Factor Receptor-Associated Factor 2 (TRAF1), and Matrix Metalloproteinase 9 (MMP-9) using the fluorescence immunoassay. The detection instrument was a KDC-12 fluorescence immunoassay analyzer (Anhui USTC Zonkia Scientific Instruments Co., Ltd., WHXB 20150107), and the detection kits were purchased from Qingdao Sankai Science & Tech Co., Ltd. (6) Comparison of Treatment Safety: The occurrence of adverse reactions such as nausea, vomiting, urticaria, drug fever, and asthma in both groups was recorded for comparison.

### Statistical analysis:

SPSS 27.0 software was used for data processing. The confidence interval was 95%. Measurement and count data were expressed as *χ̅*±*S*) and [n (%)] respectively. Meanwhile, t-tests and chi-square tests were conducted, with a significance level of α= 0.05.

## RESULTS

The overall response rate in the observation group was higher than that in the control group (*P*<0.05), as shown in Table-I. The observation group reported a shorter time to symptom subsidence than the control group (*P*<0.05) Table-II. The post-treatment levels of G-17, PG I, and PG II in the observation group were lower than in the control group (*P*<0.05) Table-III. The observation group reported higher post-treatment serum levels of GAS, VIP, and MOT than the control group (*P*<0.05), as shown in Table-IV.

**Table-I:** Comparison of clinical response rate [n (%)].

Group	n	Significantly Effective	Effective	Ineffective	Overall Response Rate
Observation	58	30(51.72)	23(39.66)	5(8.62)	53(91.38)
Control	58	25(43.10)	20(34.48)	13(22.41)	45(77.59)
*χ^2^*					4.209
*P*					0.040

**Table-II:** Comparison of the time to symptom subsidence/ulcer wound healing (d, `c±s).

Group	n	Subsidence of stomach pain	Subsidence of bleeding	Subsidence of bloating/belching	Ulcer wound healing
Observation	58	8.41±1.72	10.19±2.92	15.12±3.65	17.58±4.92
Control	58	10.18±2.45	13.67±3.34	18.23±4.01	21.63±5.34
*t*		4.503	5.973	4.368	4.248
*P*		0.000	0.000	0.000	0.000

**Table-III:** Comparison of gastric mucosal function indicators (`c±s)

Group	n	G-17(ng/ml)		PG I(ng/ml)		PG II(ng/ml)	
		Pre-treatment	Post-treatment	Pre-treatment	Post-treatment	Pre-treatment	Post-treatment
Observation	58	296.21±30.23	182.31±19.42^*^	211.52±23.45	141.32±15.39^*^	89.44±9.12	44.14±5.92^*^
Control	58	298.54±31.62	199.45±17.83^*^	214.03±23.22	158.92±16.42^*^	88.54±8.14	49.63±6.14^*^
*T*		0.258	4.951	0.579	5.956	0.561	4.902
*P*		0.796	0.000	0.564	0.000	0.576	0.000

**Table-IV:** Comparison of gastrointestinal hormone levels (`c±s).

Group	n	GAS (ng/ml)		VIP (ng/ml)		MOT (mg/ml)	
		Pre-treatment	Post-treatment	Pre-treatment	Post-treatment	Pre-treatment	Post-treatment
Observation	58	109.23±14.14	181.44±16.91^*^	92.45±12.31	191.22±18.05^*^	242.52±27.43	362.34±37.43^*^
Control	58	108.36±13.13	160.01±18.92^*^	93.25±11.45	180.39±19.42^*^	244.41±28.19	321.63±31.62^*^
*t*		0.343	6.431	0.362	3.111	0.366	6.328
*P*		0.732	0.000	0.718	0.002	0.715	0.000

The post-treatment levels of TRX-1, TRAF1, and MMP-9 in the observation group were lower than in the control group (*P*<0.05) Table-V. No significant differences were observed in incidences of adverse reactions between the two groups (*P*>0.05) Table-VI.

**Table-V:** Comparison of inflammatory factor levels (`c±s).

Group	n	TRX-1 (ng/ml)		TRAF1 (ng/ml)		MMP-9 (ng/ml)	
		Pre-treatment	Post-treatment	Pre-treatment	Post-treatment	Pre-treatment	Post-treatment
Observation	58	80.25±8.09	44.35±5.29^*^	121.23±13.34	68.34±7.41^*^	41.54±5.16	19.45±3.41^*^
Control	58	79.62±7.98	50.32±5.54^*^	123.03±14.45	74.33±8.16^*^	42.65±5.72	23.28±3.89^*^
*t*		0.422	5.936	0.697	4.139	1.097	5.639
*P*		0.674	0.000	0.487	0.000	0.275	0.000

**Table-VI:** Comparison of treatment safety [n (%)].

Group	n	Nausea/vomiting	Urticaria	Drug fever	Asthma	Overall Incidence
Observation	58	2(3.45)	2(3.45)	2(3.45)	1(1.72)	7(12.07)
Control	58	2(3.45)	1(1.72)	1(1.72)	1(1.72)	5(8.62)
*χ^2^*						0.372
*P*						0.542

## DISCUSSION

In this study, the observation group received a treatment intervention combining antibacterial drugs with proton pump inhibitors and bismuth, and the specific medications included amoxicillin capsules, rabeprazole enteric-coated capsules, and bismuth potassium citrate capsules, resulting in satisfactory effect and significantly improved overall response rate in patients.^10^ This is attributed to rabeprazole, a classic proton pump inhibitor, which primarily works by binding to the H+/K+-ATPase (proton pump) on the membrane of gastric parietal cells, inhibiting its activity and thus reducing gastric acid secretion.^11^ Meanwhile, rabeprazole can reduce gastric acid-induced erosion on the ulcer surface by decreasing gastric acid secretion, thereby creating a favorable environment for healing.^12^

Additionally, rabeprazole promotes the repair and regeneration of gastric mucosa, accelerating ulcer healing. Moreover, rabeprazole exhibits some antibacterial effects against Hp, directly attacking the urease of Hp and inhibiting its growth.^13^ Therefore, rabeprazole exerts a beneficial therapeutic effect on Hp-infected peptic ulcers through multiple mechanisms. Bismuth potassium citrate is a commonly used gastric mucosal protectant that binds to the mucus and proteins on the surface of gastric mucosa, forming a protective gel-like substance^14^ that covers the ulcer surface, preventing further erosion of the gastric mucosa by gastric acid and pepsin, and also inhibits pepsin activity, thereby protecting the gastric mucosa.

Moreover, this drug enhances the mucus barrier function of the gastric mucosa, promoting its repair and regeneration, which is beneficial for accelerated ulcer healing. However, clinical applications have shown that the combination of rabeprazole and bismuth potassium citrate exhibits limited efficacy against Hp-related peptic ulcers, particularly regarding its antibacterial effects on Hp. To tackle this issue, amoxicillin, an antibacterial drug sensitive to Hp, was added to the observation group for oral treatment. Amoxicillin belongs to the β-lactam class of antibiotics, the antibacterial mechanism of which involves binding to transpeptidases during bacterial cell wall synthesis, inactivating the enzyme, and disrupting the pathway for cell wall synthesis.

Consequently, bacterial cells rupture and dissolve due to changes in osmotic pressure, leading to cell death.^15^ Clinical observations indicate a satisfied antibacterial activity of amoxicillin against Hp, as well as its low resistance rate, especially when used in combination with proton pump inhibitors and bismuth. Specifically, proton pump inhibitors enhance the antibacterial effect of amoxicillin by inhibiting gastric acid secretion and increasing the gastric pH. This combined medication regimen significantly improves the eradication rate of Helicobacter pylori, resulting in effective treatment outcomes for Hp-infected peptic ulcers, with a shorter time to symptom subsidence than that in the control group.^16^

The progression of peptic ulcer disease is accompanied by changes in gastric mucosal function indicators. G-17, a gastrointestinal hormone secreted by G cells in the gastric antrum and fundus, has elevated levels closely associated with atrophic gastritis, gastric ulcers, and Hp infection. PG I primarily reflects the function of gastric acid-secreting cells, with an increase in gastric acid secretion leading to elevated PG I levels, while PG II mainly reflects the condition of gastric fundic mucosal lesions, where an increase in PG II indicates pathological states such as atrophy of gastric fundic glands and gastric epithelial metaplasia.^17^

The observation group exhibited lower levels of G-17, PG I, and PG II than the control group, indicating that the combined treatment regimen improved gastric function in patients. This improvement is attributed to the synergistic effect of three medications, that is, rabeprazole, which inhibits gastric acid and improves the gastric environment, bismuth potassium citrate, which protects the gastric mucosa and facilitates the repair of damaged mucosa, and amoxicillin, which enhances the antibacterial effect against Hp, contributing to the repair of damaged gastric mucosa and ultimately restoring gastric function.^18^

Due to symptoms such as belching and acid reflux, patients with Hp-infected peptic ulcers often experience gastrointestinal dysfunction, leading to abnormal changes in gastrointestinal hormone levels. GAS, VIP, and MOT are common gastrointestinal hormone indicators. Specifically, GAS, secreted by G cells, stimulates gastric and intestinal motility, VIP, a peptide neurotransmitter released by intestinal neurons, relaxes intestinal smooth muscle and reduces gastric retention, and MOT, a critical gastrointestinal hormone, promotes intestinal peristalsis and accelerates the passage of intestinal contents.^19^

In this study, the post-treatment levels of GAS, VIP, and MOT in the observation group were higher than those in the control group, suggesting that the combined treatment regimen can improve gastrointestinal hormone levels in patients. The occurrence of inflammatory responses is also a significant reason for the progression of peptic ulcers, characterized by the abnormal expression of various inflammatory factors during the onset of the disease. Serum TRX-1, an antioxidant protein associated with oxidative stress damage, has been found to exhibit high expression in patients with peptic ulcers, which can enhance the activity of hypoxia-inducible factors in gastric mucosal cells and promote cell damage. In the meantime, TRAF1, an essential member of the tumor necrosis factor family, binds specifically to its ligand TNF, activates downstream signaling pathways that promote cell survival, proliferation, and differentiation, and contributes to inflammation and immune regulation processes, showing high expression in patients with peptic ulcer. Additionally, MMP-9, a member of the matrix metalloproteinase family, is secreted by macrophages, monocytes, and neutrophils, contributing to the development of inflammatory responses.^20^

Moreover, the observation group reported lower post-treatment levels of TRX-1, TRAF1, and MMP-9 than the control group, benefiting from the strong anti-inflammatory effects of rabeprazole and amoxicillin included in the combined treatment regimen, which helps mitigate the levels of inflammatory responses. Furthermore, the safety assessment indicated no severe adverse reactions resulting from the addition of amoxicillin in the observation group, suggesting the safety of the combined treatment regimen.

### Limitations

However, this study also has some shortcomings like a small sample size and no follow-up was conducted. In view of this, more samples should be included and follow-up time should be increased in future studies to further validate the findings of this study.

## CONCLUSIONS

In conclusion, the combination of antibacterial drugs with proton pump inhibitors and bismuth is significantly effective in treating Hp-infected peptic ulcers by promoting symptom subsidence, improving gastric function, enhancing gastrointestinal hormone levels, inhibiting the expression of inflammatory factors, thereby demonstrating satisfactory treatment safety.

### Authors’ Contributions:

**XF:** Conceived, designed the study and Review, is responsible and accountable for the accuracy or integrity of the work.

**QS:** Collected the data and performed the analysis. Critical review.

**YJ:** Collected the data, performed the analysis, critical review, were involved in the writing of the manuscript. All authors have read and approved the final manuscript.
